# Successful respiratory management of a Marshall-Smith syndrome patient with a tracheo-innominate artery fistula

**DOI:** 10.1186/s40981-020-00343-6

**Published:** 2020-05-22

**Authors:** Satoko Noguchi, Junichi Saito, Jun Kawaguchi, Tetsuya Kushikata, Kazuyoshi Hirota

**Affiliations:** Department of Anesthesiology, Hirosaki University Graduate School of Medicine, 5 Zaifu-cho, Hirosaki, Aomori, 036-8562 Japan

**Keywords:** Marshall-Smith syndrome, Tracheo-innominate artery fistula, Difficult airway management

## Abstract

**Background:**

Tracheo-innominate artery fistula (TIF) is a life-threatening complication of tracheostomy. We describe perioperative management for innominate artery transection in a case with TIF.

**Case presentation:**

A 4-year-old Japanese female with Marshal-Smith syndrome presented for management of TIF. She underwent tracheostomy at the age of 3 months and an uncuffed tracheostomy tube was inserted. One month before admission to our hospital, intermittent tracheal bleeding, suggesting TIF, occurred. Although we considered to change to a cuffed endotracheal tube, craniofacial abnormality suggested difficult oral intubation, and there was a possibility of rebleeding. Finally, innominate artery transection was performed under total intravenous anesthesia without changing the tracheostomy tube. Surgery completed uneventfully and she received mechanical ventilation under sedation for a day, followed by weaning without complications.

**Conclusions:**

A cuffed tracheostomy tube should have been inserted before surgery for effective hemostasis against sudden bleeding from TIF even though conversion to oral intubation was difficult.

## Background

Marshall-Smith syndrome (MSS) is a rare genetic disorder described by Marshall et al. in 1971 [1]. MSS is caused by mutations in the NFIX gene [2]. In patients with MSS, difficult airway associated with unique facial features such as a high forehead, exophthalmos, prominent premaxilla, and retrognathia associated with accelerated skeletal maturation is one of the major concerns for anesthesiologists [3, 4]. In addition, severe respiratory dysfunction that may be present in a patient with MSS can lead to a severe clinical course in the neonatal or early infant periods.

Long-term MSS survivors have recently been reported, due to early diagnosis and successful interventions such as tracheostomy and positive pressure ventilation. However, several potential complications are associated with long-term survival in MSS. We herein report a patient with MSS who underwent innominate artery transection for trachea-innominate artery fistula (TIF) following tracheostomy, and we discuss the respiratory management of such patients during the perioperative period.

A written consent was obtained from the patient’s guardian to publish this case report.

## Case presentation

Our patient was a 4-year-old Japanese female who underwent an urgent innominate artery transection under general anesthesia due to a suspected TIF. She was born at 37 weeks’ gestation with a natural delivery. Her birth weight was 2542 g and the APGAR scores were 7 and 8 at 1 and 5 min, respectively. At birth, the patient was immediately intubated due to forced breathing and hypoxemia. Her craniofacial anomalies including exophthalmos, prominent forehead, blue sclera, and micrognathia indicated the possibility of one or more complications of a genetic disease. Radiographic examinations demonstrated advanced skeletal maturation, and she was diagnosed MSS at 2 months old. She underwent tracheostomy for tracheomalacia at 3 months old, followed by gastrostomy at the age of 2 years, but not required ventilator support. Gastrostomy was performed under inhalation anesthesia via tracheostomy tube under spontaneous breathing in combination with regional anesthesia.

**Fig. 1:**
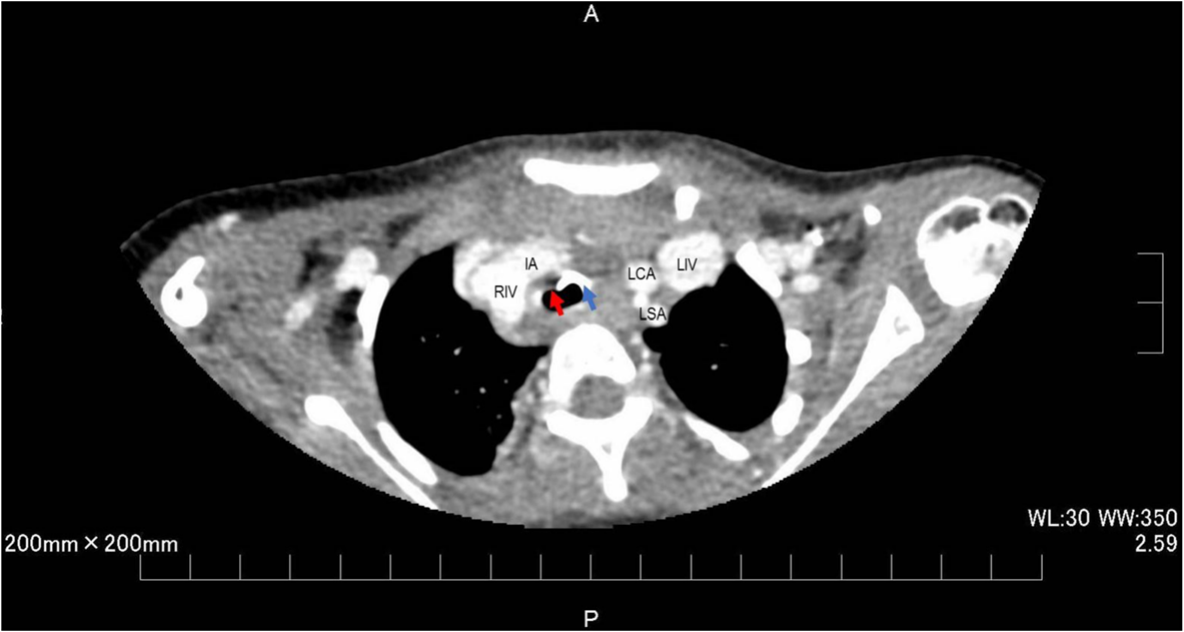
Preoperative contrast-enhanced computed tomography. Computed tomography demonstrated the innominate artery was in contact with the trachea. Red and blue arrow shows tracheo-innominate artery fistula and the tip of cannula, respectively. IA; innominate artery, RIV; right innominate vein, LCA; left common carotid artery, LSA; left subclavian artery, LIV; left innominate vein

One month before the patient’s admission to our hospital, intermittent tracheal bleeding was observed and managed by a change in the smaller cannula size and shorter length. Two weeks later, the patient needed hospitalization at a primary medical clinic because of acute tonsillitis and pneumonia-induced hypoxemia. Intermittent tracheal bleeding reappeared, and granulation at the distal site of the cannula without bleeding was detected by bronchoscopy. Contrast-enhanced computed tomography revealed extremely thin connective tissue between the innominate artery and the trachea, suggesting TIF and she was transferred to our hospital (Fig. 1).

After admission, the patient’s vital signs were stable; blood pressure 96/57 mmHg, heart rate 143 bpm, and SpO_2_ 97% (room air attached with heat and moisture exchangers). Her height and body weight were 81 cm and 8 kg, respectively. A 4.0 mm ID uncuffed tracheostomy tube of a length that was customized for the patient was used, and air leakage during bag valve ventilation was limited. There was a risk of surgical site infection and postoperative mediastinitis due to the short distance between the cannula and surgical site, but we decided not to change the tracheal cannula because the patient’s hypersalivation and craniofacial abnormality predicted difficult oral intubation, and there was a possibility of rebleeding due to inadvertent granulation stimulation.

**Fig. 2:**
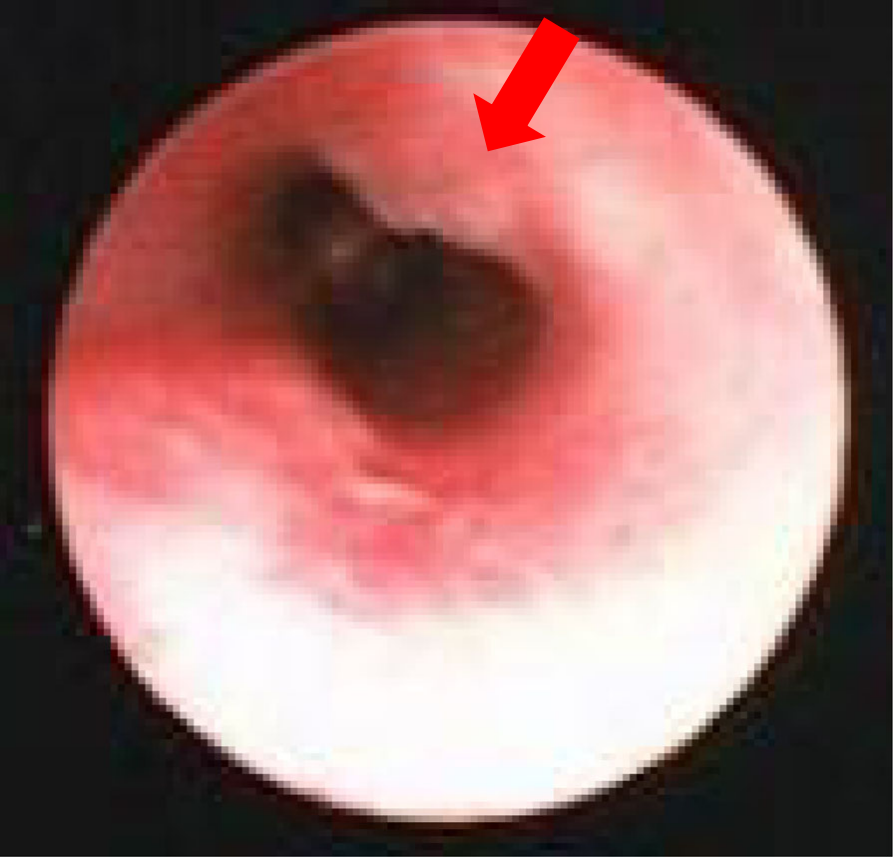
Preoperative bronchoscopy findings. Bronchoscopy after the induction of anesthesia showed a white ulcer lesion (→) at the anterior trachea

Anesthesia was induced and maintained with midazolam, ketamine, and fentanyl. Rocuronium was administered to prevent coughing during surgery. In addition to the standard ASA monitoring, the invasive blood pressure (left radial artery), central venous pressure (CVP) (right femoral vein), and regional cerebral oxygen saturation (rSO_2_) at the sites of the head and back were monitored during anesthesia. No specific response occurred during the induction of anesthesia; blood pressure 80/43 mmHg, heart rate 145 bpm, SpO_2_ 100%, CVP 5 mmHg, rSO_2_ values 95/53, and pharyngeal temperature 36.5 °C. Bronchoscopy after the induction of anesthesia showed a white ulcer lesion at the anterior trachea (Fig. 2).

The patient was first placed on pressure-controlled ventilation, but the end-tidal CO_2_ increased to around 60 cmH_2_O after rocuronium administration because of air leakage through the vocal cords. Although a tube exchange to a cuffed tube was considered, there was no cuffed tracheostomy tube that would fit her, and it was difficult to perform even the oral bronchoscopy due to the limited oral space. We thus placed oral pharyngeal gauze to decrease the air leakage, and the patient's end-tidal CO_2_ then gradually decreased to around 45 cmH_2_O.

Median sternotomy was performed followed by clamming of the innominate artery at the proximal and distal sites in contact with the trachea. Although the regional cerebral oxygen saturation temporarily decreased by 10% from the control value when the innominate artery was clamped, other vital signs were nearly stable and the innominate artery was transected without any graft replacement.

A bronchoscopy after the operation revealed no evidence of active bleeding in the patient's trachea, and she was transferred to the intensive care unit (ICU). To prevent surgical-site bleeding and unexpected movement, 0.2 mg/kg/h midazolam and 0.4 μg/kg/h dexmedetomidine were administered until postoperative day 1. Spontaneous respiration was detected 1 h after admission to the ICU, and the pharyngeal gauze was then removed. After discontinuation of the sedatives, consciousness recovered to the preoperative level and the ventilation management was also ended. Although frequent aspiration was required due to hypersalivation, no additional treatment for hypoxemia was needed. The patient was discharged to a general ward on postoperative day 2, and she was transferred to her previous clinic on postoperative day 8.

## Discussion

Total intravenous anesthesia and dexmedetomidine were safely used for this MSS patient during the perioperative period, with no complications. Due to the poor prognosis of MSS, the anesthetic management of MSS patients is rare; the experiences of sedation and general anesthesia have been reported for only five cases of MSS (Table 1) [3–7]. No prolonged action or complications were observed in those cases [3–7]. Inhalation anesthesia with sevoflurane alone enabled management under spontaneous respiration for an orthopedic minor surgery [4], and a combination of remifentanil with sevoflurane provided adequate reflection suppression for fiber intubation via a laryngeal mask [7]. These reports suggested the effectiveness of inhalation anesthesia with a short-acting opioid (e.g., remifentanil and fentanyl) under minor surgery for prompt recovery.

**Table 1:** Previous case reports of anesthetic management for MSS

Case	Reference	Age, sex	Height, weight	Type of surgery	Airway management	Premedication	Anesthetics	Muscle relaxant	Complication
1	Antila et al. [3]*	7 m, M	71 cm, 6.85 kg	Tympanostomy	Blind intubation	n.a.	Thiopental Fentanyl	None	Hypoxia Bradycardia
2		8 m, M	71 cm, 6.85 kg	Bronchoscopy	Spontaneous	n.a.	Ketamine Diazepam	None	None
3	Watanabe et al. [4]	4 years, M	99.6 cm, 13.5 kg	Trigger finger	Mask-assisted	None	Sevoflurane Nitrous oxide	None	None
4	Salik et al. [5]	17 y, M	n.a., 44 kg	Ophthalmic surgery	Via tracheostomy	n.a.	Midazolam Fentanyl Sevoflurane DEX	Rocuronium	None
5	Nakano et al. [6]	10 years, M	115 cm, 24 kg	Tooth extraction	Intubation with McGrath	Midazolam	Propofol Fentanyl Sevoflurane	Rocuronium	None
6	Machotta et al. [7]	9 m, M	n.a., 8.5 kg	Ophthalmic surgery Gastrostomy	Intubation with FS and LMA	n.a.	Sevoflurane Remifentanil	Cisatracurium	None

*DEX* dexmedetomidine, *FS* fiberscope, *LMA* laryngeal mask airway, *n.a.* not available

*Cases 1 and 2 were the same patient, and case 2’s surgery was 2 weeks after the case 1 surgery. A literature search was performed using “Marshall-Smith syndrome” as search term in PubMed

However, another concern for MSS patients is postoperative agitation after moderately invasive surgery. As osseous fragility and profound intellectual disability have been reported in MSS patients [8], it is important to prevent postoperative agitation. It is also necessary to avoid an increase in secretions due to postoperative excitement, including those due to respiratory complications and the influence of wound infection. Sevoflurane has been reported to be associated with an increased risk of emergence agitation [9]. And the management of total intravenous anesthesia avoided poor sedation by leakage of inhalation anesthesia during bronchoscopy. In our patient’s case, we therefore used total intravenous anesthesia for the surgery and then sedated her with dexmedetomidine during the postoperative period in the ICU; this protocol provided adequate sedation with no complications.

Hemorrhage from a TIF is a rare but fatal complication of tracheostomy [10]. Although there was no airway hemorrhage during our patient’s surgery, it was necessary for the surgeons to have a preoperative discussion about how to manage bleeding and airway issues such as a tracheal tube exchange in the surgical field and the induction of percutaneous cardiopulmonary support. The efficacy of temporary hemostasis achieved by over-inflating a tracheostomy tube during surgery has been reported [11]. Regarding the efficacy of hemostasis against sudden bleeding from a TIF, it may be better to change to an endotracheal cuffed tube preoperatively.

Risk factors of TIFs have been reported: tracheal infection, steroid use, creation of the tracheostomy below the third tracheal ring, pressure necrosis caused by overinflation of the cuff or malposition, and chest deformity leading to a high-riding innominate artery [10]. The neck of some MSS patients is relatively short with delayed head skeletal growth, and this often requires a relatively lower-level tracheostomy. It is thus not surprising that MSS patients may have a TIF.

## Conclusions

In conclusion, we report the successful anesthesia management of an MSS patient with a TIF who underwent an urgent innominate artery transection with no respiratory complications.

## Data Availability

Please contact corresponding author for data requests.

## Abbreviations

CVP: Central vein pressure
ICU: Intensive care unit
MSS: Marshall-Smith syndrome
rSO_2_: Regional cerebral oxygen saturation
TIF: Tracheo-innominate artery fistula
